# Enhancing industrial acoustic environments through a mathematical model and 3D COMSOL acoustic simulation

**DOI:** 10.1038/s41598-026-42609-6

**Published:** 2026-03-31

**Authors:** Amira M. Eladly, Nabil Rashwan, Moustafa H. Aly, Wessam M. Salama

**Affiliations:** 1 College of Engineering, Pharos University in Alexandria, Alexandria, Egypt; 2 Arab Academy for Science Technology and Maritime Transport College of Engineering and Technology, Alexandria, Egypt

**Keywords:** Industrial noise reduction, 3D acoustics simulator, COMSOL multiphysics, Machine placement, Spinning industry, Engineering, Mathematics and computing

## Abstract

Legislators and manufacturing clients often drive engineering firms to adopt noise reduction measures early in community projects. This paper introduces a mathematical model and simulation tools to analyze sound propagation impacts at acceptable distances by optimizing machine placement based on sound engineering principles. A 3D acoustic simulation program, employing a diffusion equation model, forecasts noise distribution levels before and after machine layout modifications using a genetic algorithm. Our study took place in a spinning factory, where the optimized machine layout improved indoor acoustic conditions from 91.22 dB to 88.17 dB. This framework effectively reduces the daily work exposure level (LEX, 8 h) in the textile industry by 3.344%.

## Introduction

The impact of disease and mortality due to environmental pollution poses a challenge for public health, especially in developing countries. Before the 1990 s, it was typical for governments and scientific research to prioritize solid waste, water, and air pollution. In reality, a working group of the World Health Organization (WHO) concluded in 1971 that noise poses a major threat to people health; according to the National Institute for Occupational Safety and Health (NIOSH), noise is deemed detrimental to human health when its decibel level exceeds 85 dB^1^. It may be unable to block out these sounds while operating in an area where such disturbances frequently occur. Industry regulations state that people close to noise sources of pollution work near sources that generate ear-splitting noises^2^.

In industrial settings, workers are often exposed to significant amounts of noise. These noisy environments are a health hazard that can cause temporary or permanent hearing loss and may also cause stress and fatigue. Protecting workers in such settings can be challenging. Companies are required by law in many countries to protect their workers from exposure to excessive noise. Reducing noise to safe levels without compromising the productivity or efficiency of a process requires planning and can be challenging.

The textile sector has experienced rapid growth in raw materials, industrialization, and job opportunities. Increasing automation in the textile industry and the evolution of mass production techniques are the main causes of high noise levels. The work period was extended to 8 h per day. The highest noise level the employees are subjected to is 92,8 dB, and the lowest noise exposure threshold is 86.9 dB^3^. By carefully arranging the noise source under the spatial distribution of the noise level, the impact of noise on the workers can be controlled.

The paper’s main contribution is an optimal spatial distribution derived from mathematical modeling that minimizes Sound Pressure Levels (SPLs). It presents a computational strategy for noise reduction using effective predictive techniques. Although the proposed solutions can be experimentally validated with physical instruments, manual measurements are impractical given the size of the spinning machines and the production process’s effects on subsequent studies. A carefully selected noise simulation model predicts noise distribution in the plant, aiding in the validation of the mathematical model’s outcomes. The 3D acoustic simulation was performed using COMSOL Multiphysics software, utilizing the diffusion equation module to create various noise emission scenarios. AutoCAD was employed to model the spinning facilities, providing essential geometric and semantic information, such as equipment dimensions, locations, materials, and noise emission data, thus effectively simulating noise conditions.

The main objective of this paper is introduced as follows:

- The paper presents a mathematical approach to optimally arranging spinning machines to reduce noise and minimize SPLs within the facility.
- It offers a predictive method for assessing and enhancing noise levels through a noise reduction strategy.
- A 3D acoustic simulation model using the diffusion equation module in COMSOL Multiphysics is developed to predict noise dispersion and validate the mathematical model’s predictions.
- Additionally, AutoCAD is employed to realistically simulate noise by modeling the spinning facility’s geometric and semantic features, including machine dimensions, locations, materials, and noise emissions.
- Ultimately, these techniques provide a scalable solution for effectively simulating and analyzing noise environments, addressing the limitations of manual noise measurement and machine layout testing constrained by size and manufacturing processes.

This paper is organized as follows: Sect. 2 reviews studies on noise mapping, simulation, and noise in the spinning industry. Section 3 details the proposed methodology. Section 4 presents an example that illustrates and evaluates the methodology, including results and a performance discussion. The conclusion with suggestions for future work are presented in Sect. 5.

## Literature review

### Noise mapping and simulation modelling in industry

Industrial spaces are often noisy, which can cause discomfort, stress, or injury. An acoustic model of the facility is useful for noise investigations during licensing. Additionally, post-commissioning adjustments can enhance the acoustic model to reduce noise levels.

According to Putro et al.^4^, the study of noise mapping in the industrial field aims to accomplish four main goals: identifying appropriate noise monitoring technology; visualizing and monitoring noise pollution levels; preparing mitigation plans for potential expansion or before building new facilities in the same industrial complex; and appropriately selecting hearing protection devices/equipment for its employees. Squadrone et al.^5^ utilize acoustic data from equipment manufacturers to update noise modeling in industrial plants transitioning from operation to design phase, marking the final step in optimizing sound source balance. A calculated noise map was developed based on design modifications, aligning with the observed map to assist in equipment maintenance, restoration planning, and the establishment of comparable new plants.

Computer programs visualize noise distribution in two or three dimensions through color-coded maps indicating noise intensity. In a spare parts factory in Caxias do Sul, Brazil, the Sound PLAN software was used to predict noise emissions and model indoor sound propagation by analyzing and measuring SPLs within the facility^6^.MATLAB R2013 (8.1.0.604) and Surfer Vs 13 software were utilized to create noise maps for the thermal power plant in Mbalbayo, Cameroon^7^. The noise study in the Dis Industrial Zone of Ümraniye, Istanbul, Turkey, employed the noise mapping programs Sound PLAN ISO-9613-2 and Lden. A 3D model of the area was generated from altitude readings in AutoCAD format and imported into Sound PLAN alongside a digital terrain model (DTM)^8^.

COMSOL Multiphysics has been widely used in recent industrial research. For instance, Song et al.^9^ utilized this finite element simulation tool to study how corridor width, carpet absorption, and classroom door size affect acoustics, aiding non-specialists in designing school buildings for acoustic assessments. Alongside safety measures, optimizing a building’s acoustic performance is crucial. Similarly, Tan et al.^10^ assessed noise impacts on maintenance staff using Building Information Modeling (BIM) and COMSOL Multiphysics, employing an optimization method to identify a maintenance schedule that minimizes daily noise exposure. This approach has led to an enhanced maintenance plan for offshore platforms, prioritizing noise impact in safety management.

### Noise in spinning industry

High-speed machines essential for spinning raw fiber into yarn generate significant noise, typically ranging from 80 to 100 dBA. Research in Bombay indicates an average noise level of approximately 96.5 dBA. Equipment like carding machines, drawing frames, ring frames, and winding machines consistently produce noise too high for an 8-hour workday. Studies show that over 30% of spinning workers are frequently exposed to noise levels exceeding 90 dBA^11^. Many employees choose to work longer hours for additional pay, adversely affecting their health and psychological well-being. According to Norman et al.^12^, 32.1% of spinning factory workers suffer from noise-induced hearing loss. This study aims to mitigate the negative health impacts on workers by enhancing the acoustic environment in spinning factories, thereby promoting safety and improving productivity while ensuring compliance with industrial regulations.

Insulation materials are often used to reduce noise levels. Arjunan et al.^13^ developed a finite element model to assess sound insulation in metal-framed walls, studying the geometric impact of stud frames on acoustic performance. The model effectively visualizes noise behavior in advanced and multi-material structures.

The most recent study to use the technique of changing the positions of sound sources to reduce the overall noise level was conducted by Lan in 2007^14^. An Exterior Penalty Function Method (EPFM) model optimized equipment allocation and noise reduction, assessed using a 16-station system. The Environmental Noise Model (ENM) verified results and created noise contour maps. EPFM minimized deviation from the target 60 dB(A) SPL. Optimized designs reduced boundary noise to below 60 dB(A), as confirmed by noise contour maps.

Therefore, this study aims to address the gap in research concerning the redistribution of noise sources to reduce SPL.

## Methodology

A spinning plant in Borg El Arab City, Egypt is implemented in this paper, known for producing high-quality compact yarns with counts (Ne) ranging from 40 to 140, including fine and superfine types. The production process consists of eight key steps: Blow Room Line, Carding, Draw Frames, Combing Preparation, Combing, Roving Frames, Compact Spinning, and Winding. The spinning hall is notably spacious, measuring 40 m wide and 122 m long.

**Fig. 1:**
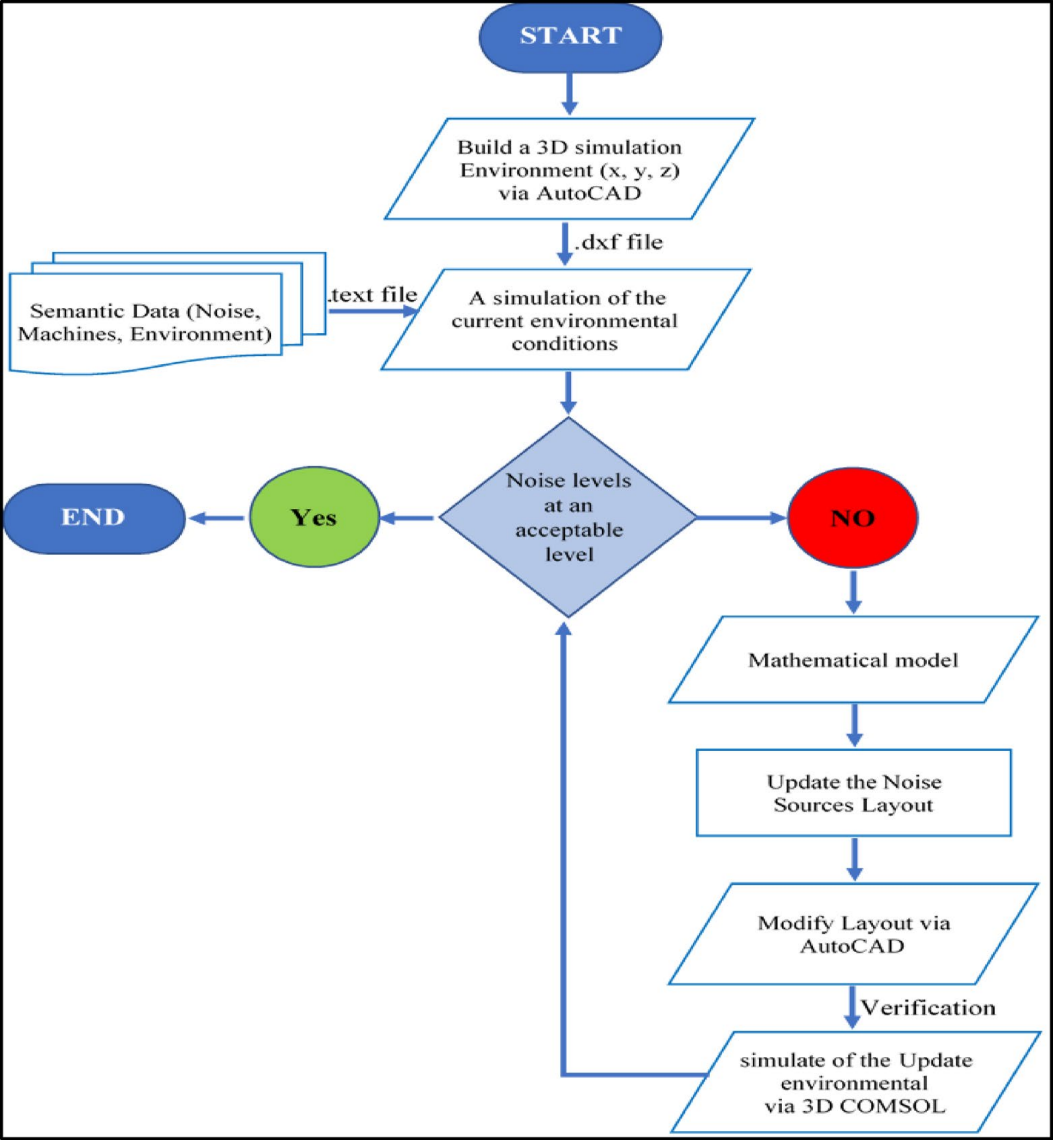
Flowchart of the proposed framework.

Figure 1 illustrates the flowchart of the proposed framework, which showcases a 3D simulation environment established via a Live Link between AutoCAD and COMSOL Multiphysics. Current environmental conditions were simulated by importing semantic data from text files into COMSOL. A mathematical model was developed based on the arrangement of noise sources and simulation data for noise emissions. The original and modified layouts are compared and evaluated through reconstruction and testing using COMSOL.

Figure 1 illustrates the iterative, decision-driven methodology used to refine the layout. The flowchart outlines the steps involved.

Step 1: Setting the noise simulation environment in COMSOL Multiphysics.
  A 3D model (x, y, and z coordinates) of the spinning production hall is built via AutoCAD and imported into COMSOL.
  Simulation parameters, loaded from a.txt file, included spatially varying sound speed ${c}$ and energy density ${w}$ based on machine materials, noise emission statistics, absorption coefficients for cotton, walls, ceiling, and floor, and impedance boundary conditions (absorption or reflection).
  The boundary condition is set, as well as the area of the sound source for each piece of equipment.
  Given the diverse acoustic properties of the simulation areas, the acoustic diffusion equation (ADE) model is chosen for its precision and computational efficiency in industrial settings. A finer mesh is used for greater accuracy.
Step 2: Noise modeling or computation.
  The noise levels and identifying areas of concentration upon execution are calculated.
  Noise metrics are computed if it accepts the target criterion.
  The program ends if the input industrial area noise levels are acceptable; otherwise, a mathematical model is employed to improve noise reduction.
Step 3: Decision diamond.
  Yes branch: “Noise levels are at an acceptable level.” Leads to END, indicating a satisfactory layout has been achieved.
  No branch: a mathematical model is employed to improve noise reduction. Triggers a refinement loop through multiple processing steps, then rechecks noise levels.
Step 4: Mathematical Modeling.
  Governing equations: The acoustic field is described by the Helmholtz equation in the frequency domain:
  Boundary conditions: Material interfaces and boundaries are modeled with impedance or absorbing conditions to accurately capture reflections and losses.
  Material properties and geometry: Spatially varying properties (speed of sound, density, and impedance) and geometric parameters capture the design features (e.g., plate orientations, chamber volumes, and perforations).
  Objective and constraints: Define a quantitative noise metric, weighted SPL over a region or total radiated sound power) and impose fabrication, weight, and size constraints as part of the optimization problem.
  Sensitivity information: Derive adjoint or gradient information to enable efficient design updates.
Step 5: Layout Adjustment and Optimization.
  Design variables: Geometric parameters (dimensions, orientations, and boundary shapes) and material choices that influence the acoustic response.
  Design objective: Formulate a composite objective that minimizes deviation of the simulated noise metric from a target level while penalizing undesired attributes (cost, weight, manufacturability).
  Optimization strategy: Use gradient-based methods when adjoint sensitivities are available; otherwise, employ surrogate models or heuristic optimization (e.g., Bayesian optimization).
  Manufacturability constraints: Enforce minimum feature sizes, tolerances, and assembly compatibility to ensure feasible implementations.
  Geometry generation: Produce updated geometries corresponding to the optimized design variables for subsequent simulation.
Step 6: Simulation the update environment (Decision diamond-2).
  If acceptable: finalize the layout (END).
  If not acceptable, adjust the layout and repeat the evaluation loop until the noise target is met.

### Data collection

As mentioned earlier; a fin compact yarn is produced in a 40 m by 122 m rectangular spinning hall through eight manufacturing processes.

Measurement laboratories are located adjacent to the production hall, separated by a glass partition. Based on these measurements, background noise levels measured under non-production conditions are 38.59 dB.

Table 1 details the machinery and equipment for each process, categorized by production section, quantity, and model. To enhance understanding, including a brief description of each machine’s primary function would be helpful. The sound pressure levels (SPL) for various equipment range from 77 to 86 dB, indicating significant noise exposure that exceeds the standard occupational safety limit of 85 dB for an 8-hour workday per OSHA regulations. Therefore, workers must wear appropriate protective gear and implement noise reduction measures. Additionally; information on the sound source area and machine dimensions (height, width, and length) is important for noise modeling and spatial planning.

Comprehending sound travel within the facility requires noting the area of the sound source, such as 8.6989 m² for carding machines. It’s important to clarify whether this area pertains to the acoustic influence or the machine footprint. Given their arrangement, the nine carding machines, producing 79–82 dB SPL, significantly contribute to the overall noise level. Despite only having two larger machines with a sound source area of 13.858 m² and an SPL range of 77–84 dB, they also add to the noise. The Small Spinning Devices area stands out as the primary noise source, featuring the largest sound source area (74.4207 m²) and 15 units with an SPL of 83–86 dB. The necessity for targeted noise control is underscored by another sound source area of 10.535 m² with an SPL of 82–85 dB. The chart emphasizes the importance of implementing noise control strategies, particularly in sections like Compact Spinning and Winding, suggesting solutions such as improved machine enclosures, acoustic barriers, or designs that minimize overlapping noise sources.

**Table 1:** Production machine data, and (SPL) for each section.

Production section	No of units	Machine model	Sound pressure level (SPL)		Dim in m			Area of ​​sound source
			dB	mW	Length	Width	Height	
Carding machines	9	RIETER C70	79–82	0.3162	3.325	2.38	3.655	8.6989
Draw frame m/c	2	RIETER SB-D45c RSB-D45c	77–84	0.1	4.1	3.38	1.775	13.858
Combing preparation m/c	5	RIETER E34 OMEGAlap	82–85	0.3162	4.833	1.798	2.176	8.68973
Combing m/c	5	RIETER E80	83–85	0.3162	8.152	2.438	3.733	19.8746
Roving frames m/c	3	RIETER F16	80–82	0.1	33.45	1.859	2.2	62.1836
Compact spinning m/c	15	RIETER K46	83–86	0.3162	70.076	1.062	2.005	74.4207
Winding machines	15	SAVIO Polar I-DSL	82–85	0.2512	9.92	1.062	3.15	10.535

**Fig. 2:**
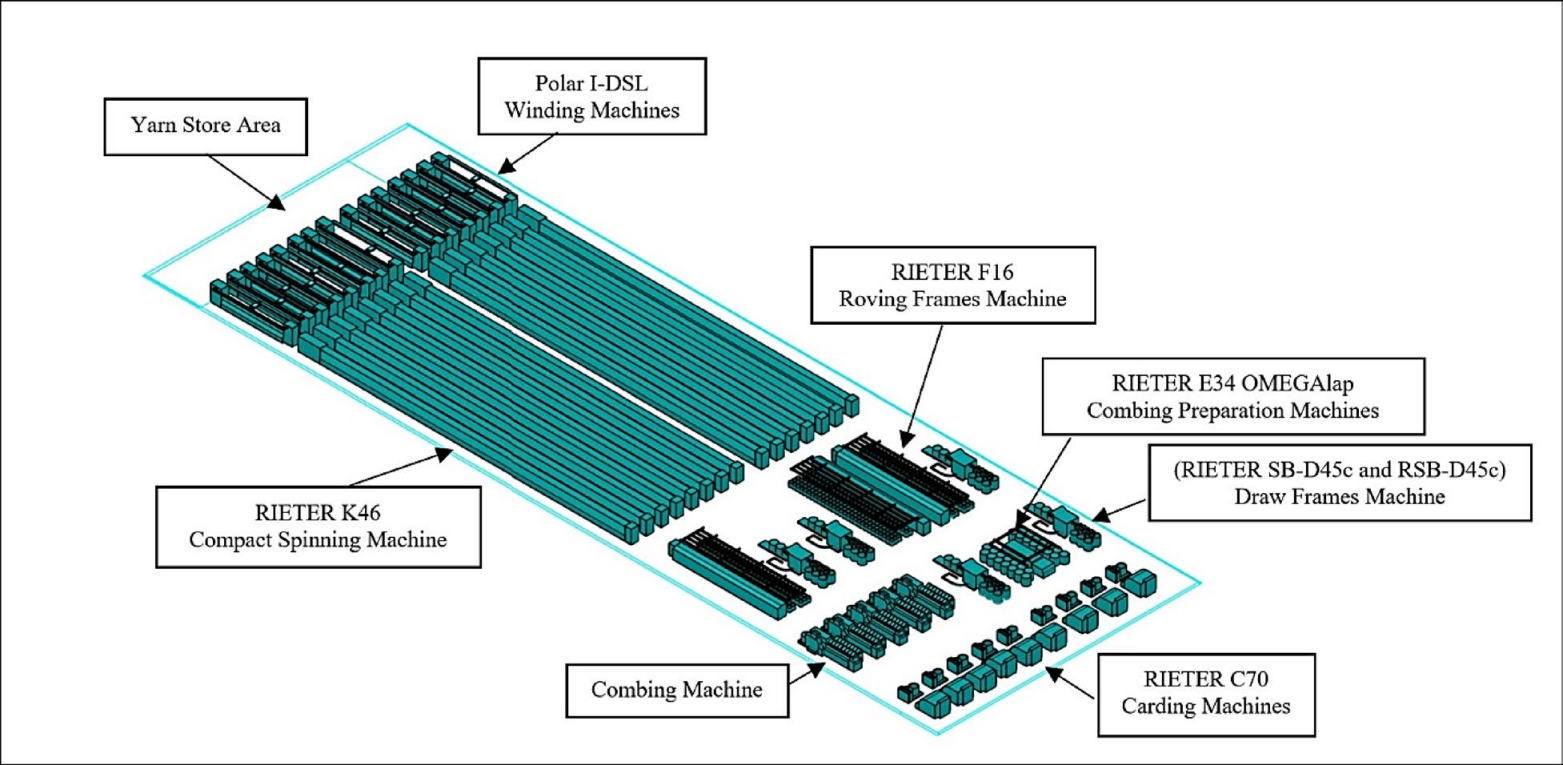
The machine arrangement in the spinning hall.

To facilitate the simulation software for the paper environment, the 3D AutoCAD application constructs the geometric layout and dimensional data of the machines and equipment in the spinning hall across the x, y, and z axes for each operational module, as shown in Fig. 2.

The configuration of the machines in the spinning hall is clearly illustrated in Fig. 2’s 3D view, with labeled production divisions facilitating the identification of machine locations. The yarn store area is distinctly marked, providing perspective on the facility’s operations. Central to the layout, the small spinning machines (RIETER K46) highlight their significance in the production process. The Polar I-DSL winding machines are placed near the yarn store, likely optimizing yarn storage post-processing. The arrangement of other equipment, including carding (RIETER C70) and combing preparation (RIETER E34 OMEGAlap) machines in separate zones, suggests a logical workflow.

### Setting the noise simulation environment in COMSOL Multiphysics

#### Acoustic simulation physics module

Industrial areas feature various acoustic characteristics influenced by their large volumes, which are typically longer and wider than their tall. These spaces often contain obstacles like benches, material piles, and machinery that scatter and absorb sound waves. Consequently, the acoustic environment changes significantly when the area is occupied versus when it is empty, leading to non-diffuse reverberated sound fields. As a result, few approaches exist to solve the propagation equation^15^. Billon et al.^16^ suggest that a diffusion equation-based numerical model effectively addresses most issues in these acoustic spaces, such as atmospheric attenuation, aperture coupling, wall transmission, and sound absorption by scattering objects. Thus, the acoustic diffusion equation (ADE) model was chosen for its high precision and reasonable computational cost in acoustic simulations, outpacing other models.

#### Construct the geometry and define the material properties

The machine 3D layout was created using AutoCAD software, simplifying the geometric features compared to the original. Implementing the original detailed functional modules directly could be costly. The constructed geometry was imported via Livelink for simulation access. Air was chosen from the built-in library as the study domain. Simulation environment parameters were loaded from a.txt file that included materials for the machines and equipment, noise emission statistics for each machine, and the noise absorption coefficients for cotton, walls, ceiling, and floor as illustrated in Table 2^17^.

**Table 2:** Materials’ noise reduction coefficients (NRC) values.

Material		Noise reduction coefficient (NRC)	Material		Noise reduction coefficient (NRC
Walls	Brick unpainted. Gypsum board, 12 mm on studs.	0.05	Floors	Hardwood. Heavy carpet on concrete.	0.3
	Concrete block. Expanded polystyrene 50 mm battens.	0.35		Wood floor boards.	0.15
	Wood paneling, 25 mm with air gap.	0.1		Solid wooden floor.	0.1
	PU foam 6–50 mm thick.	0.35–0.9		Parquet on concrete.	0.05
	Thin carpet with no underlayment.	0.2		PU foam 6–50 mm thick.	0.35–0.9
	Plywood panel 3 mm thick.	0.01–0.02		Thin carpet with no underlayment.	0.2
	Wood wool cement 25 mm battens.	0.6–0.7			
Ceilings	Mineral wool 100 mm thick.	0.65	Windows	Single pane glass 3 mm thick. Double glazing 2–3 mm, 1 cm air gap.	0.05
	Mineral Fiber tile 19–25 mm thick.	0.75–0.85		Generic Fiber glass tile 25 mm thick.	0.85
	Fiber glass board 16 kg/m3, 25–100 mm thick:	0.7–1.2		Fiber glass board 16 kg/m3, 25–100 mm thick.	0.7–1.2
Others	Cotton panel 48 kg/m3, 25–50 mm thick:	0.8–1.15 A wide NRC range indicates variability based on thickness, density, installation, and mounting.		Window glass.	0.15
	Bare Metal/Machine Body.	0.00–0.10		Fiber glass board 48 kg/m3, FRK 25–50 mm thick:	0.65–0.75

#### Boundary condition and mesh setting

This study examined the working environment of the spinning hall, located in a rectangular area with six boundaries, designated as the computational domain. Each piece of machinery and equipment has a sound source area, as outlined in Table 1. The Acoustic Diffusion Equation (ADE) interface, part of the COMSOL commercial finite element solver, solves acoustic energy density distribution in room acoustics. This physics interface effectively models noise distribution in enclosed spaces.

The ADE model is governed by Eq. (1):

1 $$\frac{{\partial w}}{{\partial t}} + \Delta .J + cm_{a} w = q$$

where ${w}$ is the sound energy density, ${c}$ is the sound speed (default 343 m/s, variable by environment), ${{m}}_{{a}}$ is the absorption from airborne losses (determined by the volume absorption coefficient), ${J}$ represents local energy flux (W/m²), and vector q is the spatial sound source. The local energy flow to the noise source is embodied in ${J}$.

Accurate COMSOL acoustic simulations require optimized meshes balancing resolution and memory. Use physics-controlled meshes with frequency-dependent element sizes, boundary layers, and Perfectly Matched Layers (PMLs).

For pressure acoustics, maintain element size ≤ λ/5 to λ/6 (λ = smallest wavelength), using hexahedral or mapped meshes near boundaries and boundary layer meshing at walls. Employ swept meshes in PMLs for absorption. For high frequencies, adjust mesh sizes across frequency bands or consider the Boundary Element Method (BEM).

Mesh density levels range from very coarse to extremely fine, with local/custom options for user-defined control.

Advantages include high accuracy, memory efficiency via anisotropic boundary layers, reduced reflections via PMLs, and Multiphysics capability. Disadvantages include high computational cost, frequency limits necessitating dense meshes, complex setup, and potential convergence issues with anisotropic elements.

**Fig. 3:**
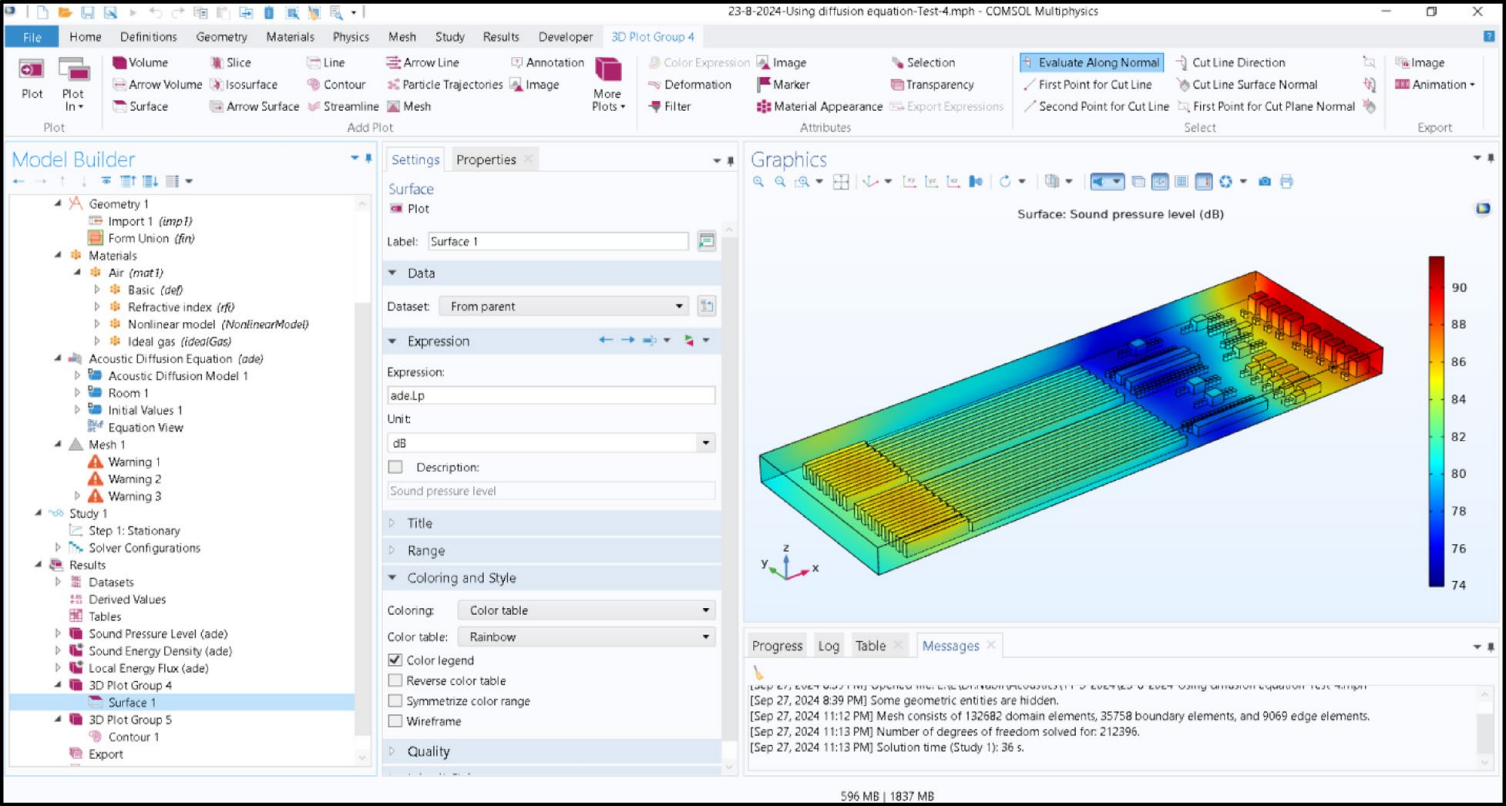
3D COMSOL interface for the proposed environment.

To solve the model, the mesh used for its geometry is crucial. Each mesh cell provides a separate solution to the equation, which, when integrated across the entire network, yields a comprehensive solution. A finer mesh was used for greater accuracy, but the large study area made the extra fine mesh unnecessary and expensive. Additionally, the results were identical/similar. The COMSOL interface used in this study is illustrated in Fig. 3.

### Mathematical model for noise reduction and layout optimization

The proposed model aims to create a mathematical framework that minimizes noise and optimizes equipment layout to reduce sound propagation impact over specified distances. We tackle this by integrating sound engineering principles with distance optimization techniques. The key variables include machine locations, distances, and SPL, focusing on the following aspects:

- Organizing equipment and surfaces to reduce walking distances and workflow disruptions while maximizing space between high-noise sources.
- Applying the inverse square law, which states that sound pressure level decreases with distance, necessitating the placement of noisy equipment as far from sensitive areas as possible.
- Implementing acoustic panels or walls as sound barriers to deflect or absorb noise.
- Arranging low-noise equipment in clusters when distance considerations are less critical.

#### Minimizing noise (SPL)

The noise in an environment is typically measured using the SPL, in dB. For multiple sources, the combined SPL can be calculated using:

2 $${SPL}_{total}=10.{\mathrm{log}}_{10}(\sum_{i=1}^{N}{10}^{SPL/10})$$

- $N$ is the number of machines.
- ${SPL}_{total}$is the sound pressure level of the machines.

Our objective is to minimize ${SPL}_{total}$while ensuring a minimum distance between machine positions.

#### Distance optimization

To minimize the distance between machines, we can use the Euclidean distance formula:

3 $${d}_{ij}=\sqrt{{({x}_{i}-{x}_{j})}^{2}+{({y}_{i}-{y}_{j})}^{2}+{({z}_{i}-{z}_{j})}^{2}}$$

where ${d}_{ij}$​ is the distance between the machine $i$ and machine $j$, $\left({x}_{i},{y}_{i},{z}_{i}\right)$ are the coordinates of the machine $i$, and $\left({x}_{j},{y}_{j},{z}_{j}\right)$ are the coordinates of the machine $j.$.

- Objective function:
  We aimed to find a configuration that minimizes both ${SPL}_{total}$ and the sum of all pairwise distances while maintaining a minimum distance ${d}_{min}$ between machines, framing this as a multi-objective optimization problem.
- Incorporating the dataset:
  Using the proposed dataset, the optimization problem can be described as follows:
  $SPL$the ranges for each machine group.
  The coordinates $(x,y,z)$ of each machine.
  The minimum distances are provided for each machine pair.
- Mathematical model:
  The model is mathematically represented as follows:
  4 $$\mathrm{m}\mathrm{i}\mathrm{n}[10.{\mathrm{log}}_{10}\left({\sum}_{i=1}^{N}{10}^{\frac{SPL}{10}}\right)+\lambda.\sum_{i<j}\frac{1}{{d}_{ij}}]$$
  λ is a weighting factor that balances the trade-off between minimizing SPL and minimum distance. To establish a mathematical relationship between distance and minimum noise, we can use the inverse square law of sound propagation in free space, which states that noise levels decrease with increasing distance, as follows:
  5 $${SPL}_{new}={SPL}_{original}-20{\mathrm{log}}_{10}(\frac{{d}_{new}}{{d}_{original}})$$
  ${SPL}_{new}:$ is the new Sound Pressure Level (in dB) at a new distance ${d}_{new}$.
  ${SPL}_{original}$​ is the original SPL (in dB) at the original distance ${d}_{original}$.
  ${d}_{new}$​ and ${d}_{original}$​ are the distance between the source and the measurement point.
  To achieve a minimum noise level ${SPL}_{minumum}$ ​ given an initial distance ${d}_{original}$​ and ${SPL}_{original}$​, we can rearrange the formula.
  6 $${d}_{new}={d}_{original}.{10}^{\frac{{SPL}_{original}-{SPL}_{min}}{20}}$$

To strike a balance between practical machine spacing and noise reduction, the weighting factor is chosen. Its value is ascertained by testing several values of in a sensitivity analysis. Compact layouts with inadequate SPL reduction are preferred by lower values, but excessive spacing with slight additional noise advantages is created by larger values. From the Pareto-optimal region, an intermediate is selected, which offered efficient SPL reduction while preserving workable workflow and industrial layout restrictions.

In a parametric sensitivity analysis, all other parameters are held constant while the weighting factor is systematically changed within a normalized range (0 < ≤ 1). The optimization problem is resolved for every value of , and the resulting machine layout is assessed in terms of the necessary machine spacing and the realized SPL reduction. The sensitivity of the solution to variations in is then determined by comparing the results. Through this procedure, we are able to choose an intermediate that offers the best balance between practical layout feasibility and efficient noise reduction.

**Fig. 4:**
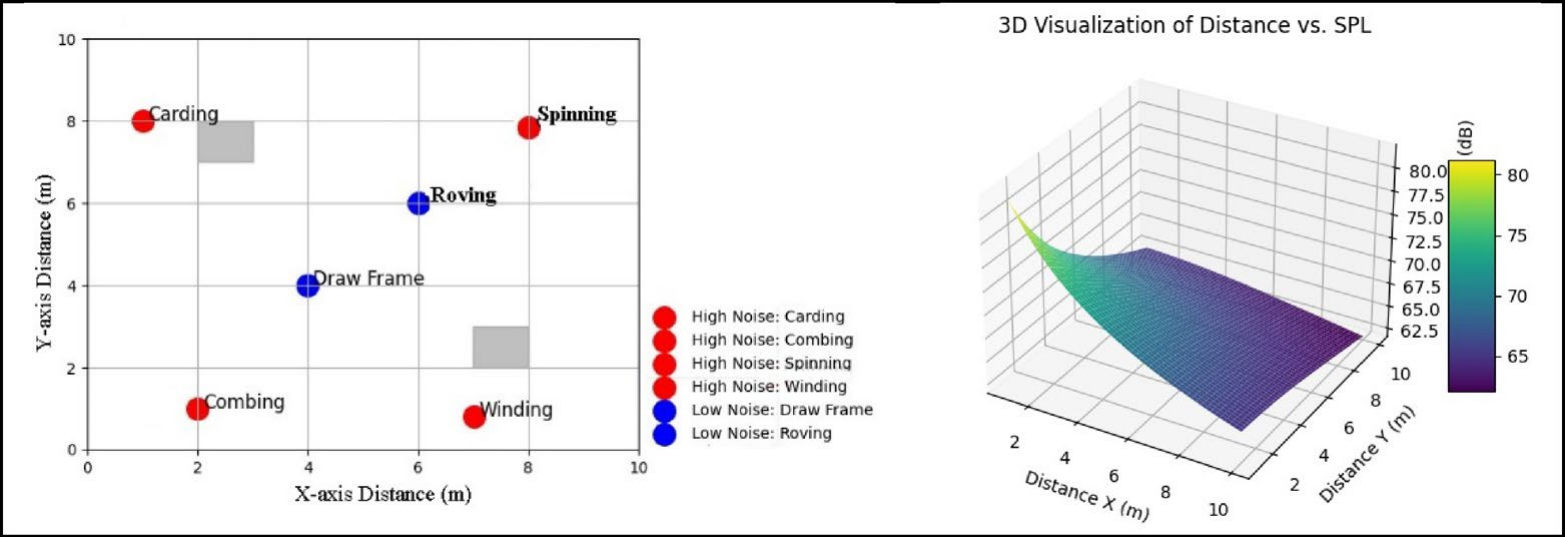
Optimized layout for minimum distance and noise.

Moreover, the weighting factor is varied throughout a normalized range in the parametric sensitivity research, for instance, = {0.2, 0.5, 0.8}. The optimizer preferred compact machine location for =0.2. A limited noise reduction (SPL reduction ≈ 1 dB) is the result of the average inter-machine distance of roughly 1.5 m. A balanced solution is found for = 0.5. By increasing the average distance to 2–2.5 m, a considerable SPL reduction of about 3 dB is achieved while still utilizing a reasonable amount of floor area. The optimization gave spacing a high priority for =0.8. The arrangement was not feasible for the factory restrictions since machine distances exceeded 3.5 m and the additional SPL decrease is negligible (only ≈ 0.5 dB improvement compared to = 0.5). These findings lead to the selection of = 0.5, which is in the Pareto-optimal region and provides efficient noise reduction without using an excessive amount of space.

d. Layout proposal:
  Figure 4. shows the manufacturing area divided into a grid by noise level, highlighting high-noise zones. The quietest machines, the draw frame, and roving machines, are marked in blue at the center, while the loudest machines, indicated in red, are located at the edges. The gray markings represent raw cotton material.

Table 3 presents a detailed analysis of the relationship between noise reduction, sound pressure level (SPL), and machine distance (M/c). The data indicates an inverse correlation between machine distance and SPL, showing that increased distance results in greater noise reduction. Specifically, SPL decreases from 85 dB at 1 m to 70 dB at 5 m, highlighting the importance of spacing for managing noise in industrial environments. Distances of 2.5 to 3 m are particularly effective, achieving a noise reduction of 10.5–12.5 dB, making them viable for facilities prioritizing noise control. While a 5-meter distance significantly mitigates noise, it may not be practical in smaller spaces due to spatial constraints.

Noise reduction increases with distance, but trade-offs include inefficient floor space usage and potential production disruptions. For small factories or confined areas, a minimum distance of 1.5 m (SPL = 80.5 dB, noise reduction = 4.5 dB) is recommended to balance noise control and functionality. When longer distances aren’t practical, supplemental noise reduction methods, such as acoustic barriers or sound-absorbing materials, can be effective. It’s also important to consider worker safety and occupational noise exposure limits. For medium-sized factories, spacing of 2 to 2.5 m offers the best balance between effective space utilization and noise reduction.

**Table 3:** The proposal machine layout replaces distance.

Distance between M/c (m)	SPL (dB)	Noise reduction (dB)	Expected noise reduction	Practical considerations	Recommended environment
1	85	0	Low	Minimal distance, risk of high noise levels	Compact spaces with limited movement
1.5	80.5	4.5	Moderate	Standard minimum distance	Small to medium factories
2	77	8	Moderate to High	The balance between space and noise reduction	Medium-sized industrial environments
2.5	74.5	10.5	High	Increased distance reduces SPL significantly	Larger spaces with room for separation
3	72.5	12.5	Very High	Significant noise reduction, reduced interference	Spacious environments, prioritizing noise control
5	70	15	Maximum	Excellent noise control, but may be impractical due to large space requirements	Very large factories or open spaces

## Results and discussion

### Noise simulation for the initial environmental

**Fig. 5:**
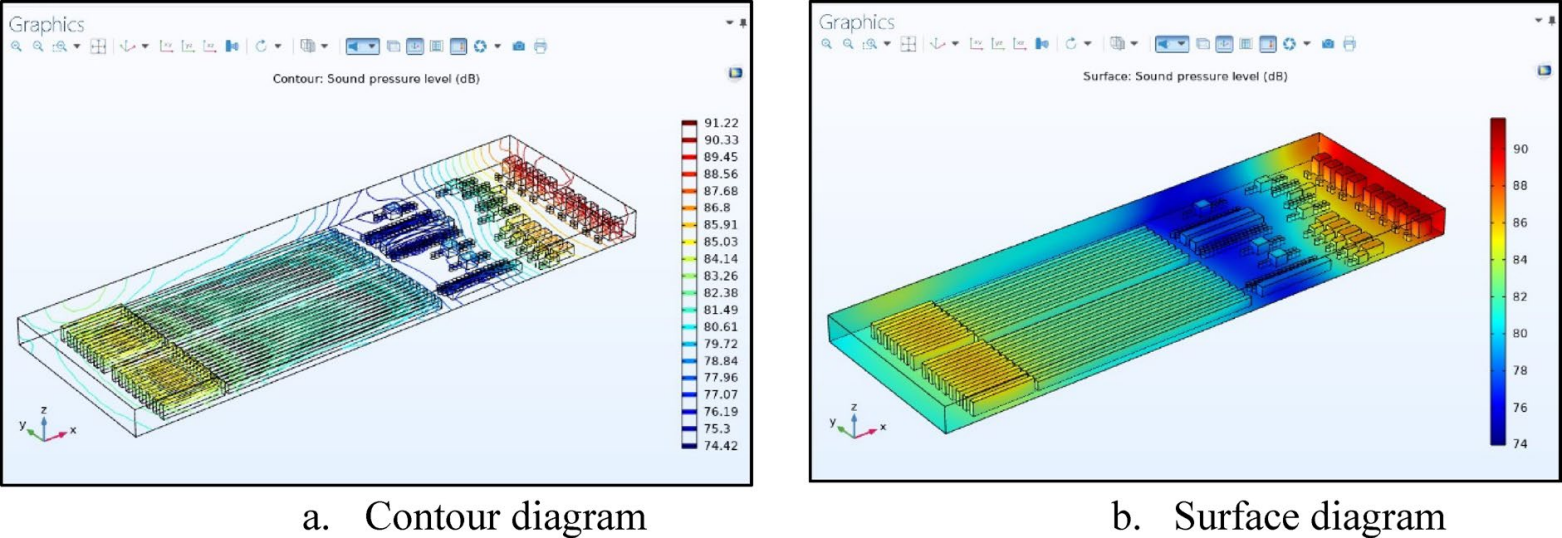
The initial environment distribution of sound pressure level (SPL) in dB. (**a**) Contour diagram, (**b**) surface diagram.

The original noise environment was assessed using a COMSOL 3D simulation, with the SPL distribution contour map shown in Fig. 5. The spinning hall’s initial process is carding, where raw fiber materials are fed into the card machine from the blowing room. This process removes impurities and aligns cotton fibers to form a “combed sliver.” The machine’s motor faces the wall, causing sound waves to reflect into the room, creating reverberation and a high noise level of 91.22 dB. The proximity of the carding machines to the wall, just 1.89 m away, and the close spacing between the machines at 1.5 m further increase the noise levels.

Following the carding machines, the combing process aligns the fibers in parallel lines, resulting in a shinier, softer, and stronger yarn. The noise levels in this area ranged from 81.49 to 88.56 dB, which are lower than those in the carding area. The next stage is the roving frame, which transforms the fibers into low-twist roving bundles ready for spinning yarn. Located in the center of the spinning hall, this stage has the lowest sound level of 74.42 dB. The decrease in noise from the combing area to the drawing area can be attributed to two factors: first, the machines’ distances—9.43 m apart for carding and combing, and 4.68 m between combing and roving. In addition, the presence of 1-m-diameter unprocessed cotton cans helps absorb sound. Cone winding is the final step of the spinning process, which involves bridging yarn production and fabric creation. Yarns are prepared for packing and advance to the weaving stage. The spinning machines, which extend 51.22 m, followed by the winding machine at 13.17 m, together comprise about 50% of the factory area. This section has sound levels between 84.14 and 85.05 dB, with the machines separated by 1.68 m and the winding machines positioned 7.34 m from the wall.

### Optimize the arrangement of the machines to reduce noise levels

The previous discussion highlighted the high average sound pressure level (SPL) in the spinning plant during working hours, underscoring the need to mitigate noise exposure for workers. A mathematical model was developed, resulting in a spatial distribution matrix for noise sources.

The mathematical model’s presumptions are directly supported by the noise reduction trends seen with greater machine spacing. Based on the inverse-square rule of sound transmission, the model explicitly assumes that SPL drops with increasing distance from the noise source. As a result, increasing machine spacing lowers SPL not just in the simulation results but also in the goal function itself. Because SPL decay is logarithmic, the model also anticipates declining returns at higher distances, which explains why noise reduction becomes less noticeable at larger spacing values. Therefore, the underlying mathematical assumptions are consistent with the observed patterns, which are a direct result of them.

Table 4 shows the relationship between distance and sound pressure levels in decibels (dB), indicating a consistent decline in SPL with increased distance from the source, which is in line with the inverse-squares law. At 1 m, the SPL is 85 dB, decreasing to approximately 47.15 dB at 20 m. At shorter distances (1 to 3.33 m) see a more rapid SPL decrease, while reductions become more gradual at greater distances. For example, SPL drops from 85 dB at 1 m to 62.74 dB at 5.26 m, resulting in a noTable 22.26-dB reduction over 4.26 m. Beyond this distance, the decline continued slowly, from 62.74 dB at 5.26 m to 52.41 dB at 9.87 m. Further reductions are minimal, such as from 50.35 dB at 11.41 m to 47.15 dB at 20 m. This plateau effect demonstrates diminishing returns for noise control as the distance increases. Achieving significant noise attenuation (22.26 dB reduction) requires maintaining a distance of about 5 m in noise-sensitive areas. Beyond this point, additional spacing results in a less noticeable reduction in SPL. The findings suggest that positioning employees at least 10 m away from noisy machinery (SPL ~ 50 dB) will foster a more comfortable and safer work environment, which is essential for meeting occupational health and safety noise exposure regulations.

**Table 4:** Correlation between the proximity of noise sources in m and sound pressure level SPL.

Distance (m)	SPL (dB)	Distance (m)	SPL (dB)	Distance (m)	SPL (dB)
1.00	85.00	7.57	56.36	14.10	48.82
1.39	82.11	7.95	55.56	14.49	48.63
1.79	79.45	8.33	54.82	14.87	48.46
2.18	77.01	8.72	54.14	15.26	48.31
2.56	74.74	9.10	53.51	15.64	48.17
2.95	72.63	9.49	52.94	16.03	48.04
3.33	70.67	9.87	52.41	16.41	47.92
3.72	68.85	10.26	51.92	16.79	47.81
4.10	67.16	10.64	51.48	17.18	47.71
4.49	65.58	11.03	51.07	17.56	47.62
4.87	64.11	11.41	50.69	17.95	47.54
5.26	62.74	11.79	50.35	18.33	47.46
5.64	61.47	12.18	50.03	18.72	47.39
6.03	60.29	12.56	49.74	19.10	47.33
6.41	59.19	12.95	49.48	19.49	47.27
6.80	58.17	13.33	49.24	19.87	47.21
7.18	57.23	13.72	49.02	20.00	47.15

An infinite number of noise emission scenarios can be calculated. When updating the machine’s positioning, it is crucial to consider workflow efficiency, energy consumption, and actual distance.

**Fig. 6:**
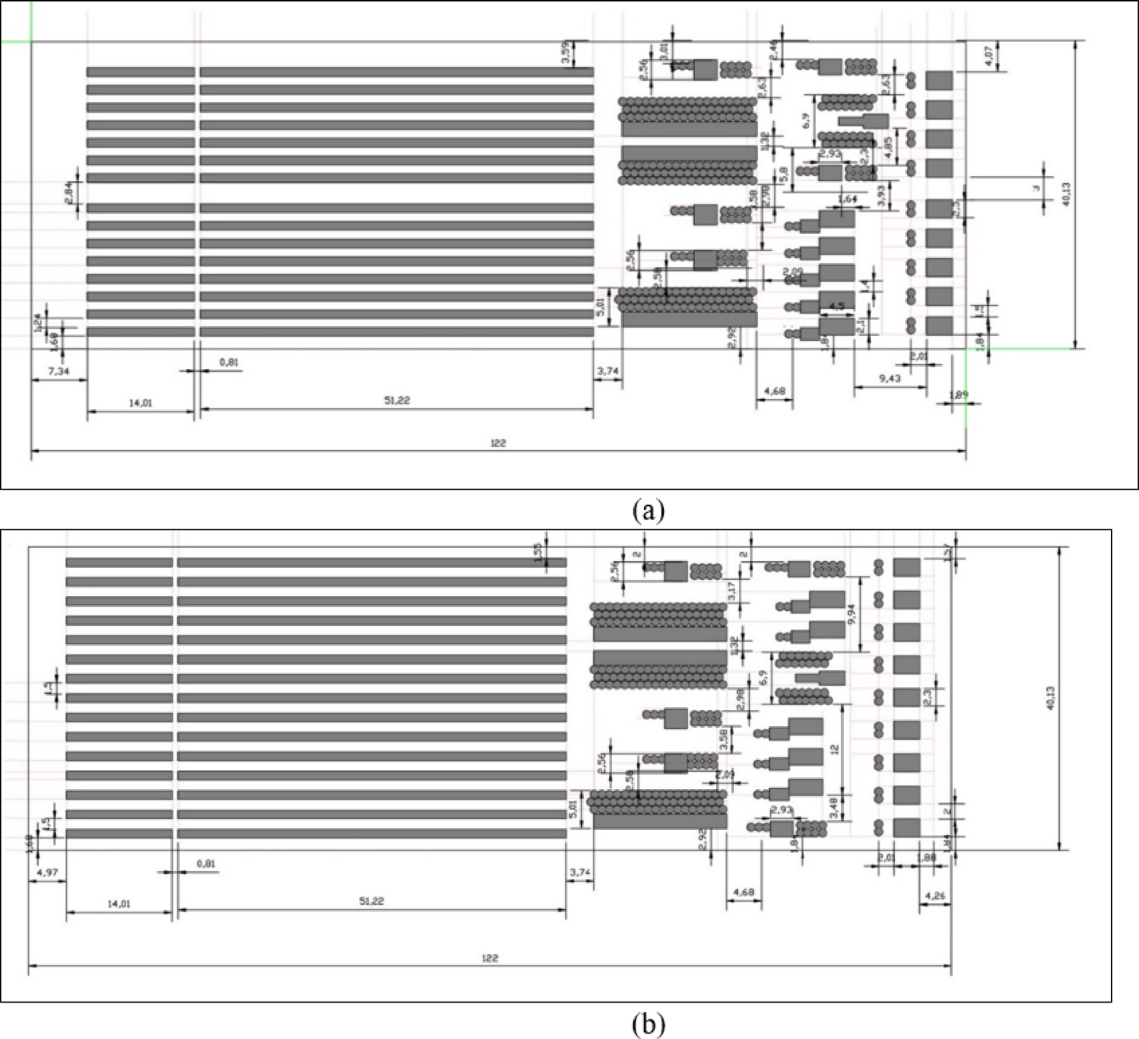
Comparison of the proposed improved machine arrangement plan with the original. (**a**) The original machine’s layout, (**b**) purpose-modified machines layout.

The spinning factory comprises three main sections: carding machines, combing and roving frames, and spinning and winding machines. In the noisy carding machine area, the distance to the wall was adjusted from 1.89 m to 4.26 m, and the spacing between the machines was increased from 1.5 m to 2 m. These changes improved the sound pressure level (SPL) from 91.22 to 88.17 dB, representing a reduction of a 2.5 dB. In the combing and roving frames section, the layout was revised in the breadth (y) direction. Initially, the first of the five combing machines was positioned 1.8 m from the wall and 1.4 m apart. The revised layout moved the two roving frame machines to the ends of the production line, maintaining a distance of 1.8 m from the walls. The three combing machines were separated by 1.9 m instead of 1.4 m, with a combing preparation machine placed between them. The average SPL decreased from 85.025 to 82.175 dB, representing a reduction of 2.85 dB. In the spinning and winding machine sections, the separation between the machines was increased from 1.24 m to 1.5 m, and the distance from the ends of the machines to the wall was modified from 7.34 m to 4.97 m. Consequently, the average SPL improved by 0.5 dB, lowering it from 81.93 dB to 81.43 dB. The original and modified machine layouts are shown in Fig. 6.

The revised design meets plant process constraints and production flow requirements. The spinning mill under study measured 40 m by 122 m. The carding area was the noisiest due to motor contact with the wall, which amplified sound reflection. The maximum significant distance change involved moving the carding machines 2.37 m further from the wall. Other modifications involved minor changes in distance relative to the factory size, including spacing the carding machines an additional 0.5 m apart. In the combing and roving frames section, the layout was revised in the breadth direction, replacing the original row of five combing machines with three machines separated by 0.5 m and a combing preparation machine placed between them. The distance between the combing machines and the wall was increased by 0.4 m, and the separation distance between machines was increased by 0.5 m. The two roving frame machines were relocated to the ends of the production line, maintaining a 1.8 m distance from the walls. The distance between the three combing machines was increased from 1.4 m to 1.9 m, a 0.5 m increase. These modifications-maintained production flow despite altering the distance between sound sources.

**Fig. 7:**
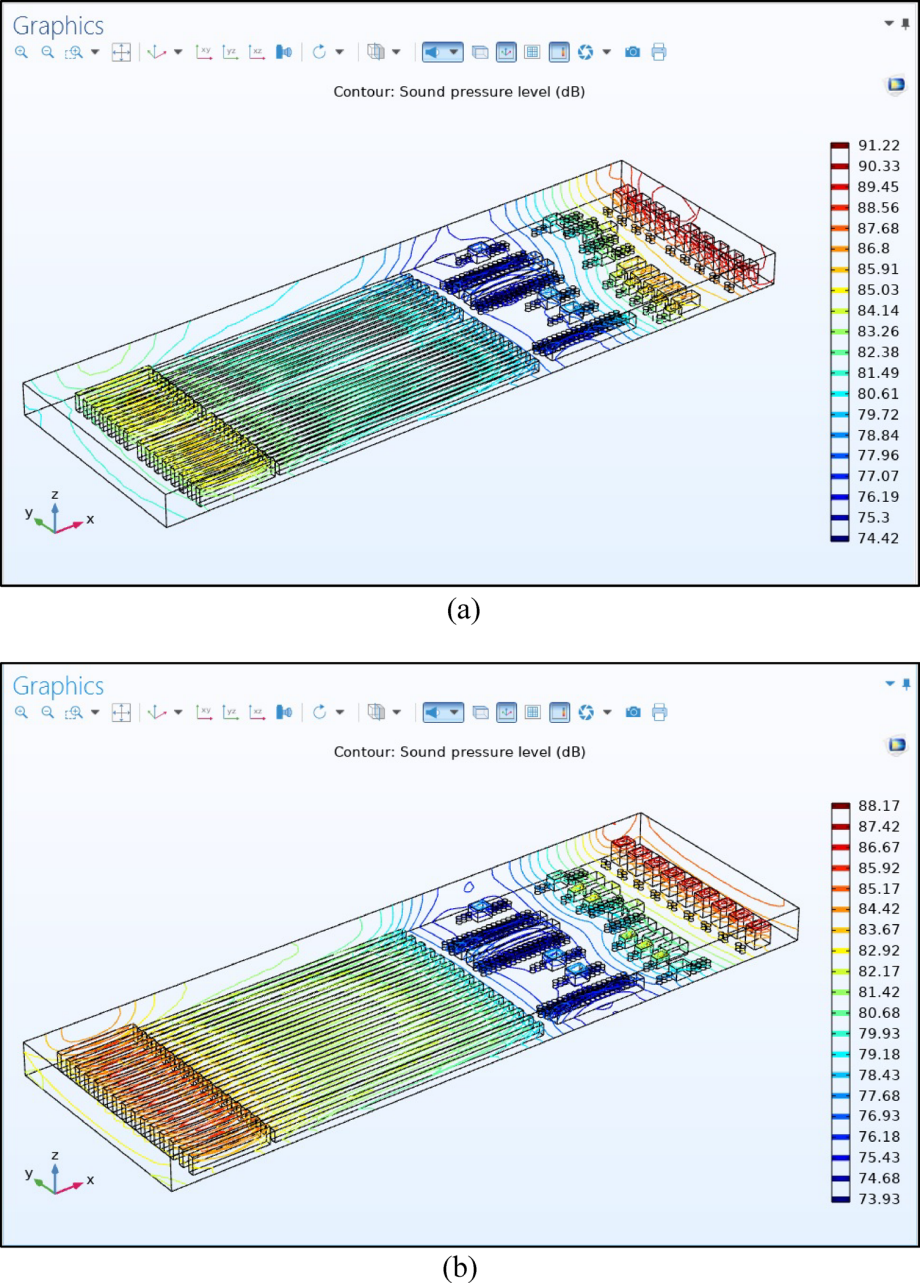
Comparison of spatial distribution and noise SPL reduction in original and modified environments. (**a**) Noise SPL in original environments, (**b**) noise SPL in the revised proposed environments.

Figure 7 shows contour maps depicting the spatial distribution of sound pressure level (SPL) on the spinning machines. As illustrated in Fig. 7a, the average SPL in the spinning factory throughout the workday is high, sometimes exceeding levels that require double pay after 8 h, leading to an unacceptably noisy environment. This highlights the need to protect workers from noise by optimizing machine configurations. Figure 7b shows an SPL reduction from 91.22 to 88.17 dB, reflecting a decrease of 3.05 dB due to the layout modifications.

Table 5 summarizes SPL changes across the main production zones.

**Table 5:** SPL changes across production zones.

Main production zones	Measured SPL (dB)	Modified SPL (dB)	Reduction value (dB)
Carding machine section	91.22	88.17	3.05
Combing and roving frames section	85.025	82.175	2.85
Spinning and winding machine sections	81.93	81.43	0.5

The contribution of individual machine groups to the overall SPL reduction is not fully isolated; due to the interconnectedness of machine groups in the open spinning industry environment, isolating the sound pressure reduction contribution of individual machine groups is challenging. Intertwined operating conditions and control strategies, including shared power sources and coordinated control loops, further complicate analysis. A holistic, system-level analysis of noise reduction is therefore more realistic than assuming separation. Since decoupling overall sound pressure reduction into distinct parts requires additional assumptions, this study investigates coordination and optimization strategies for comprehensive noise reduction jointly adjusted across different noise sources.

**Fig. 8:**
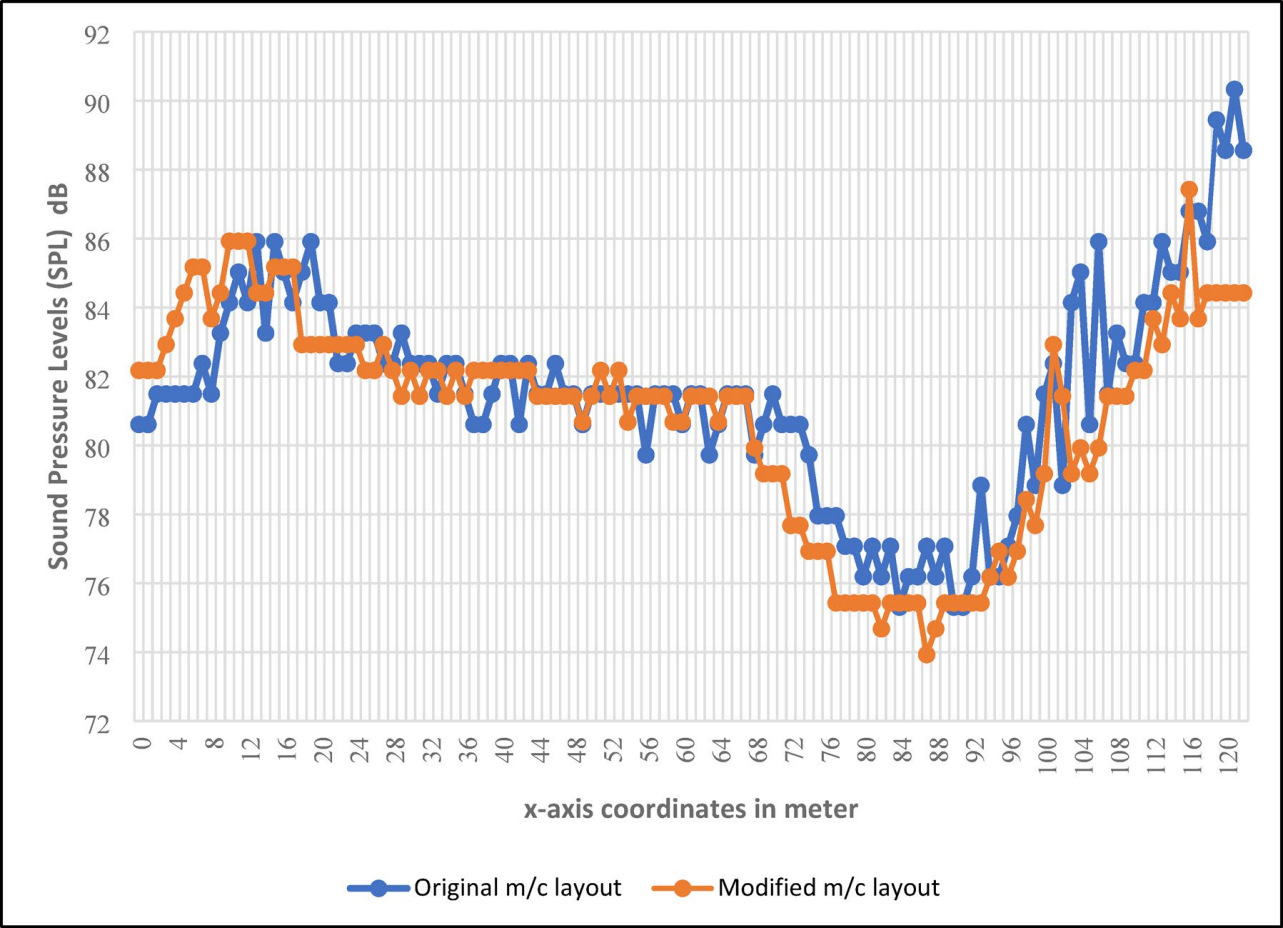
Comparison of the plant’s longitudinal distance (x-axis coordinates) in meters with the noise levels (SPLs) of the original and modified machine layouts in dB.

Figure 8 compares the noise level (SPL) in dB against the longitudinal distance of the plant in meters for both the original and modified machine layouts. Zones are defined as follows: (0–19) for Winding, (20–71) for Compact Spinning, (72–92) for Combing Spinning & Roving Frames, (93–109) for Draw Frames, and (110–122) for Carding. The figure demonstrates significant noise reduction across all zones, with the most notable reduction in the Carding zone, which is the noisiest.

The proposed 3D COMSOL Multiphysics simulation, combined with AutoCAD, accurately models noise levels in the spinning factory and visualizes the spatial distribution of Sound Pressure Levels (SPL). This enables the development of a mathematical model based on recommended noise reduction distances. However, the optimized machine layout is constrained by the production flow and minimum spacing requirements between the machines. Consequently, numerous simulation scenarios were analyzed to identify the optimal layout strategy. The proposed strategy can also be applied in other industries where high noise levels are a concern. Accurate noise simulations require updated parameters, such as reflection and absorption coefficients. Furthermore, the proposed machine arrangement strategy can be used during the design phase of spinning factories to optimize the SPL, leading to the development of improved functional modules that effectively mitigate the noise impact on workers.

Various noise reduction techniques have been applied in the textile industry, and the table below summarizes their effectiveness Table 6.

**Table 6:** Comparing noise reduction techniques.

Noise reduction technique	Reported noise reduction	Ref No
Enclosure around the drive belt and spindle	8 dB(A)	^18^
Spindle enclosure at operator position	10 dB	^19^
Isolation of spindle bearings from the holder (single spindle)	5 dB(A)	^20^
One-stage vibration isolator for high-speed spindle	9 dB	^21^
Two-stage vibration isolator for high-speed spindle	12.7 dB	^22^
Slowing spindle to ~ 70% speed (fundamental discrete tone)	≈ 9 dB reduction of the fundamental tone	^23^

Occupational exposure limits (OELs) for noise differ by country/region. For example, OSHA’s permissible exposure limit (PEL) is 90 dB (8-hour time-weighted average (TWA) with a 5-dB exchange rate)^24^, while NIOSH’s recommended exposure limit (REL) is a more conservative 85 dBA Leq (3 dB exchange rate)^25^. Egyptian law sets a maximum limit of 85 dB for 8 working hours^26^. A 3.05 dB noise reduction (from 91.22 to 88.17 dB) equates to roughly a 40–50% energy reduction. With a 3-dB exchange rate, this halves exposure energy, reducing the risk of temporary threshold shifts and noise-induced hearing loss (NIHL). While a 3-dB reduction during an 8-hour shift can lower risk and potentially achieve compliance, it might not be sufficient if OELs are exceeded, necessitating further controls.

The simulation results summarize comparative noise reduction performance across production zones as follows:

1. A 3D COMSOL simulation assessed SPLs throughout the spinning hall. The carding area exhibited the highest noise levels (SPL ~ 91.22 dB) due to motor reflections influenced by a wall distance of ~ 1.89 m and close machine spacing of 1.5 m. Noise is decreased in the combing area (81.49–88.56 dB) and is lowest in the roving frame area (74.42 dB), attributed to an increased spacing (9.43 m between carding/combing, 4.68 m between combing/roving) and sound absorption by 1 m diameter unprocessed cans. Spinning and winding zones, comprising approximately 50% of the factory area, registered SPLs in the range of 84.14–85.05 dB, with a machine spacing of 1.68 m and winding machines positioned 7.34 m from the wall.
2. Layout optimization reduces the SPL through strategic machine relocation and spacing adjustments. In the carding area, the wall distance increased from 1.89 m to 4.26 m and inter-machine spacing from 1.5 m to 2 m lowered SPL from 91.22 to 88.17 dB (2.5 dB reduction). In the combing/roving section, re-orienting roving frames to the line ends and increase spacing between combing machines from 1.4 m to 1.9 m, while maintaining wall clearances around 1.8 m, decreased average SPL from 85.025 dB to 82.175 dB (2.85 dB reduction). Increasing inter-machine spacing in the spinning/winding area from 1.24 m to 1.5 m and adjusting wall distances from 7.34 m to 4.97 m yielded a modest SPL reduction of approximately 0.5 dB (81.93 to 81.43 dB).
3. The optimized layout achieves significant SPL reductions while preserving workflow efficiency, energy consumption, and production demands. The study highlights the importance of maintaining adequate distances (e.g., around 5 m) in noise-sensitive areas for optimal noise attenuation; beyond this distance, the benefits diminish.
4. A mathematical model and spatial distribution matrix are employed to correlate distance with SPL using an inverse-square relationship. Reflection and absorption coefficients are critical parameters for accurate noise modeling and should be updated for precise predictions.

The statistical indicators can provide additional quantitative insight into the results. However, the primary objective of this study is to demonstrate the practical effectiveness of the proposed layout optimization approach in reducing overall Sound Pressure Level (SPL) under identical operating conditions.

All comparisons were conducted using the same measurement locations, boundary conditions, and source characteristics before and after optimization. Therefore, the observed reduction in overall SPL is directly attributable to the layout modification rather than variability in the experimental setup.

Moreover, the reduction trend is consistent across all evaluated receiver points, which reinforces the robustness of the optimization outcome. Since the study focuses on deterministic simulation/controlled measurement conditions rather than stochastic sampling, additional statistical variance analysis was not considered essential.

## Concusion

This work demonstrates that a combined mathematical optimization and 3D acoustic simulation framework can meaningfully reduce workplace noise exposure in a spinning factory setting. By coupling a tailored noise-attenuation model with COMSOL Multiphysics, we achieved a measurable improvement in indoor acoustic conditions: the overall SPL decreased from 91.22 dB to 88.17 dB, a 3.05 dB reduction (3.344%). Key operational levers included increasing the distance between primary noise sources (e.g., a 2.37 m increase from the carding area to the wall and 0.5 m inter-machine spacing) and reconfiguring machine distribution in the breadth (y) direction for combing and roving frames, yielding an additional 2.85 dB (3.35% SPL) reduction.

### Applicable conditions

1. The optimization assumes fixed machine types and baseline operation conditions described in the study, with the primary design variables being inter-machine spacing and spatial distribution in the x- and y-directions.
2. The acoustic model used measured source strengths for key machines and linear superposition of sound propagation in the given factory geometry.
3. The findings are most directly applicable to similar spinning/textile production environments with comparable room acoustics, layout constraints, and ventilation characteristics.
4. The workflow relies on static operating states; transient operations, startup/shutdown transients, or episodic high-noise tasks may alter SPL outcomes.

### Limitations

1. Model fidelity depends on accurate source characterization and boundary conditions (e.g., wall absorption, reflectivity, and noise-isolation features). Inaccurate inputs can bias the predicted SPL reductions.
2. The reported reductions are context-specific and may not generalize to substantially different room shapes, wall materials, or airflow patterns.
3. The study primarily targets SPL reductions and does not directly quantify effects on worker exposure duration, cognitive load, or vibration/impulse noise components.
4. Practical constraints, such as safety regulations, were not exhaustively explored in the optimization.

### Future directions

1. Extend the framework to include transient noise analysis and time-domain exposure metrics (e.g., dose, Effective Perceived Noise Level—EPNL) for occupational health assessments.
2. Incorporate noise-isolation strategies (e.g., treatment walls, barriers, and enclosure designs) and evaluate their cost-benefit trade-offs within the optimization.
3. Integrate ergonomic and safety considerations.
4. Explore multi-objective optimization that jointly minimizes SPL and energy usage (e.g., HVAC impact) while respecting spatial constraints.
5. Develop guidelines or a decision-support tool that translates optimization results into actionable layout configurations for different production lines.

## Data Availability

The data used and/or analyzed during the current study are available from the corresponding author on reasonable request.
